# Exploring the application of behaviour change technique taxonomies in childhood obesity prevention interventions: A systematic scoping review

**DOI:** 10.1016/j.pmedr.2022.101928

**Published:** 2022-07-22

**Authors:** Debapriya Chakraborty, Bronwyn A. Bailey, Anna Lene Seidler, Serene Yoong, Kylie E. Hunter, Rebecca K. Hodder, Angela C. Webster, Brittany J. Johnson

**Affiliations:** a National Health and Medical Research Council Clinical Trials Centre, University of Sydney, Camperdown, NSW, Australia; b TOPCHILD Collaboration, University of Sydney, Camperdown, NSW, Australia; c Swinburne University of Technology, Hawthorn, VIC, Australia; d School of Medicine and Public Health, The University of Newcastle, Newcastle, NSW, Australia; e Hunter New England Population Health, Wallsend, NSW, Australia; f National Centre of Implementation Science, The University of Newcastle, Newcastle, NSW, Australia; g School of Public Health, University of Sydney, Camperdown, NSW, Australia; h Caring Futures Institute, Flinders University, Adelaide, SA, Australia; i College of Nursing and Health Sciences, Flinders University, Adelaide, SA, Australia

**Keywords:** Behaviour change techniques, Obesity prevention, Children, Taxonomy, Methods development, intervention, BCT, Behaviour Change Technique

## Abstract

Behaviour change technique (BCT) taxonomies provide one approach to unpack the complexity of childhood obesity prevention interventions. This scoping review sought to examine how BCT taxonomies have been applied to understand childhood obesity prevention interventions targeting children aged 12 years or under and/or their caregivers. A systematic search was conducted in Medline, Embase, PsycINFO, Cochrane Central Register of Controlled Trials, Cochrane Database of Systematic Reviews, CINAHL and PROSPERO, to capture all eligible research up to February 2021. No limits were placed on country, language, publication dates, or full text availability. Eligible studies included any study design that applied a BCT taxonomy and evaluated behavioural childhood obesity prevention interventions targeting children aged 12 years or under and/or their parents or caregivers. Sixty-three records, describing 54 discrete studies were included; 32 applied a BCT taxonomy prospectively (i.e., to design interventions) and 23 retrospectively (i.e., to assess interventions), 1 study did both. There was substantial variation in the methods used to apply BCT taxonomies and to report BCT-related methods and results. There was a paucity of detail reported in how BCTs were selected in studies applying BCT taxonomies prospectively. Our review provides important insight into the application of BCT taxonomies in childhood obesity prevention and several ongoing challenges, pointing to the need for best practice reporting guidance.

## Highlights

• Novel critique of BCT taxonomy applications in childhood obesity prevention trials.
• Large variation in BCT methods and reporting of BCT-related methods and results.
• Scarce detail reported in selection of BCTs in prospective taxonomy applications.
• Need for guidance to standardise processes and reporting of taxonomy applications.

## 1 Introduction

The prevalence of obesity globally has reached epidemic proportions. Previously a problem of high-income countries, recently childhood overweight and obesity rates have been rising in low and middle-income countries (World Health Organization, 2021). Worldwide in 2019, 38 million children under 5 years and 340 million children aged between 5 and 19 years were experiencing obesity or overweight (World Health Organization, 2021). Early prevention of obesity in childhood is a health priority as children affected by obesity are much more likely to be affected by obesity as adults, leading to a higher susceptibility to developing chronic diseases at younger ages (e.g., hypertension, cardiovascular disease, insulin resistance) (World Health Organization, 2021).

Parents/caregivers and children, should be supported to prevent obesity by developing healthy energy-balance behaviours, such as positive infant feeding, optimal dietary intake, limited sedentary behaviours, sufficient activity levels and quality sleep. This review focuses on interventions at the individual level, while acknowledging the need for, and important role of interventions at an environmental and policy level (Pereira et al., 2019). Interventions for childhood obesity prevention have been studied extensively, with results showing mixed effectiveness (Brown et al., 2019, Yavuz et al., 2015, Askie et al., 2020). As the determinants of obesity are complex and varied, no single approach is likely to prevent childhood obesity (World Health Organisation, 2012). Interventions need to be systemic in nature and incorporate a variety of approaches across different settings, making them very complex (World Health Organisation, 2012). This complexity presents challenges in identifying successful or unsuccessful approaches and to upscaling or reproducing interventions, as well as to understanding important components driving behaviour change (Seidler et al., 2020).

Behaviour Change Technique (BCT) taxonomies provide a method to design or characterise interventions informed by behavioural theory principles (Michie et al., 2013). Behaviour Change Techniques are defined as “*the smallest identifiable, reproducible components of an intervention that can cause a change in behaviour”* (Michie et al., 2013). Taxonomies provide a standardised process to describe behaviour change content across interventions (Michie et al., 2018). A systematic approach to classifying the contents of behaviour change interventions via a taxonomy enables the identification of potentially effective components in both primary studies and systematic reviews (Grant et al., 2018). Of the different taxonomies available (Hoffmann et al., 2014, Abraham and Michie, 2008, Michie et al., 2011), the BCT Taxonomy v1 *(BCTTv1*) is the most comprehensive, is multi-disciplinary, and has been foundational to the progression of behaviour change science (Wood et al., 2014). By nature, application of taxonomies has a level of subjectivity (i.e., variability in how they are utilised). While training is often available, application of such taxonomies may differ based on researcher expertise, target populations, type of interventions, and level of detail in intervention descriptions.

Overall, knowledge is limited on the methods of BCT taxonomies application in research relating to childhood obesity prevention. Critique of the use and methods of applying BCT taxonomies is needed to give confidence in evidence syntheses determining effective BCTs for this population. Insight into such methods can identify best practice approaches and provide guidance for childhood obesity prevention researchers in future studies. Previous studies have assessed the impact of training on BCT coding and reporting of interventions targeting adult populations, finding both positive and mixed impacts (Wood et al., 2014, Wood et al., 2016). A previous scoping review examined the methods used by researchers to determine effectiveness of BCTs in changing health-related behaviour, however, this review did not examine the methods used to select or code BCTs (Michie et al., 2018). Consistency in the selection and application of BCTs (in prospective studies) as well as extraction and coding (in retrospective studies) is needed to 1) increase the utility of BCTs to better describe the active components of child obesity prevention intervention; 2) allow for higher quality evidence-synthesis; and 3) provide opportunity to understand the impact of specific BCTs on improving obesity-related outcomes. Following best practice application of BCT taxonomies, researchers will be able to examine BCTs that may be driving effectiveness of childhood obesity prevention interventions. This rich understanding of BCT effectiveness will allow the translation of the BCTs evidence base into quality designed and implemented interventions in practice settings.

This review sought to answer the question: *How have BCT taxonomies been applied to understand childhood obesity prevention interventions targeting children aged ≤ 12 years, and/or their caregivers?* The focus of this review was to explore the methods used by researchers in applying such taxonomies in both prospective (i.e., when designing or adapting an intervention) and retrospective (i.e., post-hoc use in reviews or secondary analyses of individual interventions) applications. Specifically, this review sought to: 1) categorise the design and key characteristics of studies that have applied a BCT taxonomy and frequency of application over time; and 2) describe the methods used in applying BCT taxonomies.

## 2 Methods and materials

This review followed a systematic approach, with a protocol prospectively registered on Open Science Framework Registries (https://osf.io/83fgw). Reporting of this review follows the Preferred Reporting Items for Systematic reviews and meta-Analyses extension for Scoping Reviews (PRISMA-ScR) checklist (Tricco et al., 2018) (Supplementary File 1). A scoping review was the most appropriate review type to investigate a broader scope of application of BCT taxonomies across both primary and secondary research, particularly given limited understanding of the breadth of literature available.

### 2.1 Eligibility criteria

Studies were eligible if they included: a) a population of children aged ≤12 years at baseline (including prenatal period) and/or their caregivers; and b) applied a BCT taxonomy in relation to childhood obesity prevention intervention(s), such as Abraham and Michie’s (Abraham and Michie, 2008) BCT taxonomy, ‘Coventry, Aberdeen & London – Refined’ (CALO-RE) taxonomy, BCTTv1 (Michie et al., 2013, Abraham and Michie, 2008, Michie et al., 2011, Michie et al., 2008) (i.e., the phenomenon of interest). Studies that included a broader population were only eligible if ≥80 % of participants were within the targeted child age group. Childhood obesity prevention interventions were defined as those aiming to change obesity-related behaviour(s), such as those relating to infant feeding (e.g., breastfeeding, formula feeding, introduction of solids), diet, movement (e.g., physical activity, sedentary behaviour), and sleep health (including sleep quality).

Any study design involving the primary or secondary analysis of childhood obesity prevention interventions were eligible. Prospective BCT taxonomy application could include study designs that report interventions, including individual or cluster randomised controlled trials, interrupted time series, quasi-randomised trials, pre-post studies, and intervention design studies with a qualitative component. Types of publications could include protocols, intervention development or primary outcome papers. Retrospective BCT taxonomy application could include reviews of interventions, secondary analyses or critiques of interventions.

Studies were excluded if they only involved children with overweight or obesity (i.e., obesity treatment), severe illness or chronic conditions that impacted their weight status or related behaviour, if they focused on prevention of stunting or underweight, or if the active intervention did not extend beyond the prenatal period.

### 2.2 Search

Systematic searches were conducted in February 2021 in Medline (Ovid), Embase (Ovid), PsycINFO (Ovid), Cochrane Central Register of Controlled Trials (CENTRAL), Cochrane Database of Systematic Reviews, and Cumulative Index to Nursing and Allied Health Literature (EBSCO). Unpublished studies were searched for via the International Prospective Register of Systematic Reviews (PROSPERO) and indirectly via CENTRAL, which includes registration records from the WHO International Clinical Trials Registry Portal and ClinicalTrials.gov. Targeted searches of clinical trials registers were beyond the resource constraints for this review (Aromataris, 2020). The search strategy included keywords relating to the population of interest (e.g., ‘child’, ‘infant’, ‘parent’), and application of a BCT taxonomy (e.g., ‘behaviour change technique’), in the context of child obesity prevention (e.g., ‘obesity’, ‘overweight’). There were no restrictions on country, language, publication dates, or full text availability. The full search strategy is included in Supplementary File 2. Reference lists of included studies were examined to identify additional eligible records.

### 2.3 Selection of sources of evidence

All titles and abstracts were screened in Covidence systematic review software (Veritas Health Innovation Melbourne Australia) by two independent reviewers. There was 92 % agreement between reviewers for title and abstract screening, and conflicts were resolved by a third reviewer. When uncertainty remained, the reference was included for full text screening. The full texts were each screened by two independent reviewers, and differences were resolved by discussion (DC, BAB, BJJ). At the full text stage there was 90 % agreement between reviewers. Forward-backward citation searches were undertaken to identify additional publications describing included studies, for example to identify a final publication where only a registration or protocol publication were retrieved in the database search. Where multiple included records described the same study, all available records were used to complete data charting.

### 2.4 Data charting process and data items

For each included study, data were extracted by one reviewer using Microsoft Excel (Microsoft Corporation, Redmond, WA), with a random 50 % sample independently verified by a second reviewer. Any disagreements that arose after data verification by a second reviewer were resolved by the senior reviewer. The data extraction tool was developed based on researcher expertise and pilot tested on a small sample of studies, then revised and finalised for data extraction. Data extraction included general study characteristics, methods and reporting of BCT taxonomy application (Table 1). Authors of one study were contacted to request Supplementary Files which could not be located online. Critical appraisal of studies was not performed as this scoping review was focused on the methods implemented, rather than evaluating results and effectiveness of interventions.

**Table 1:** Summary of data extraction tool items.

Category	Items
General study characteristics	*For all study designs:* authors geographic location publication date study design study population *For retrospective studies:* number of individual studies included
Methods and reporting of BCT taxonomy application	*For all study designs:* population target of the BCTs target behaviour/s name of BCT taxonomy BCT training undertaken by authors BCT numbers and labels total number of BCTs used/coded details of each unique BCT identified *For prospective studies:* process of how BCTs were selected *For retrospective studies:* type of information used to code BCTs such as published materials (i.e., intervention descriptions in publications) and/or unpublished materials (e.g., facilitator handbooks, participant materials) number of coders independence of coders process for managing discrepancies in coding (if applicable) how the BCT results were synthesised

### 2.5 Synthesis of results

Characteristics of included studies and applied BCT methods were narratively synthesised and presented descriptively in summary tables, to allow comparisons of key characteristics, frequency of use over time, and the BCT methods applied. Findings were summarised separately for prospective and retrospective applications of a BCT taxonomy, as while there were some common methods and reporting criteria there were several different methodological aspects related to each categories of studies. To explore commonly used BCTs across studies, unique reported BCTs were mapped against the 93 BCTs in the BCTTv1 (Michie et al., 2013), for both prospective and retrospective applications.

## 3 Results

### 3.1 Selection and characteristics of sources of evidence

**Fig. 1:**
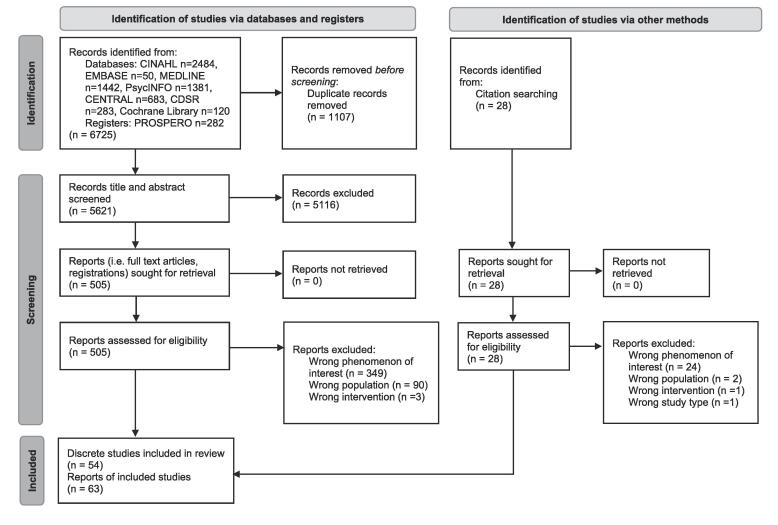
Preferred Reporting Items for Systematic Reviews and meta-Analyses (PRISMA) flow diagram of search results and selection of records.

**Fig. 2:**
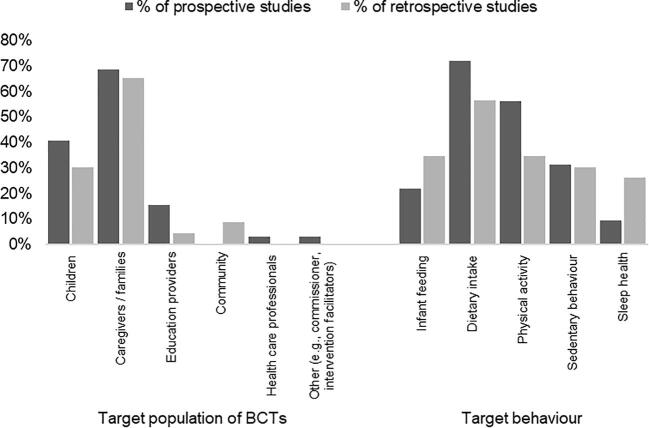
Percentage of prospective and retrospective studies^a^ by target populations and behaviours^b^. ^a^ Studies could be coded against more than one category for target population, target behaviours. ^b^ Target behaviours are grouped to infant feeding (e.g., supporting breastfeeding, appropriate formula feeding, delaying introduction of solids), dietary intake (e.g., increasing fruit and vegetables, limiting fast foods and sugar sweetened beverages), physical activity (e.g., increasing movement), sedentary behaviour (e.g., limiting screen time), and sleep health (e.g., promoting sleep routine).

A total of 6725 records were identified, with 63 records selected, describing 54 discrete studies (Fig. 1; Supplementary File 3). Of the 54 studies, results were available for 49, while 4 were registrations only and 1 was a pre-print of a protocol. Thirty-two studies were classified as prospective applications of a BCT taxonomy, most commonly of randomised controlled trial (RCT) study design (n = 16/32; including cluster-randomised and pilot RCTs), followed by studies describing intervention development (n = 7/32). Twenty-three studies were classified as retrospectively applying a BCT taxonomy. The most common retrospective study design was systematic reviews (n = 15/23); other retrospective study designs were multi-method study (n = 2/23), secondary analysis (n = 1/23), methodology study (n = 1/23), exploratory study with content analysis (n = 1/23), systematic assessment of mobile applications (n = 1/23), intervention development (n = 1/23), and scoping review (n = 1/23). The median number of primary studies assessed in the studies classified as retrospectively applying a BCT taxonomy was 16 and ranged from one (JaKa et al., 2019, Smith et al., 2018) to 64 (Webb Girard et al., 2020). One of the included studies applied a BCT taxonomy both prospectively and retrospectively as it involved adaption of an intervention to a new context (Smith et al., 2018). Regardless of prospective or retrospective BCT taxonomy application, the BCTs were most frequently targeting caregivers/families (n = 22/32; n = 15/23), followed by children (n = 13/32; n = 7/23) (Fig. 2). Dietary intake (n = 23/32; n = 13/23) was the most targeted behaviour, while sleep (n = 3/32; n = 6/23) was the least frequently targeted behaviour.

### 3.2 Methods applied in prospective use of BCT taxonomies

Table 2 presents a summary of prospective BCT taxonomy applications in childhood obesity prevention. The BCTTv1 (Michie et al., 2013) was the most frequently used taxonomy to code and develop intervention content (n = 15/32), followed by the BCT taxonomy (Abraham and Michie, 2008) (n = 8/32). The process to select BCTs was often not reported (n = 13/32) or reported with minimal description (n = 6/32). No study reported whether researchers had completed BCT taxonomy training. Approximately-one third of studies (n = 11/32) reported using an intervention design framework, stakeholders and/or evidence of BCT effectiveness to guide BCT selection. Of the studies that used the BCTTv1 taxonomy, about half reported the BCTs with numbers and labels from the taxonomy (n = 8/15).

**Table 2:** Summary of prospective BCT taxonomy applications in childhood obesity prevention (n = 32).

Features of BCT methods		Number of prospective studies^a^
**Taxonomy**	BCTTv1 (incl. adaptions)CALO-RE (incl. adaptions) BCT (Abraham et al 2008; (Michie et al., 2008) Not reported	15 5 8 4
**BCT training undertaken by coders**	BCTTv1 online training Project specific/other training Not reported	0 0 32
**Selection of BCT process**^b^	Minimal description reported Expanded description reported Adapted from an existing intervention Not reported	6 11 2 13
***Results for studies***		
**BCTs reported with number and label**	Yes No	8 24
**Average number of BCTs reported per study**^c^**(n = 15)**	Studies reporting BCTTv1: Median 14 Range 6 to 30 per study	

a Prospective study examples include randomised controlled trials, intervention development studies.

b Minimal description of BCT selection process includes only mentioning theory or behavioural determinants guided selection; Expanded description of BCT selection process includes reporting using an intervention design framework, stakeholders, evidence of BCT effectiveness etc.

c Note this does not differentiate different target behaviours, types or intensity of interventions.

Most studies were published by researchers in the UK (n = 9/32), USA (n = 8/32), and Australia (n = 7/32). The remainder in Ireland (n = 2/32), New Zealand (n = 2/32), China (n = 1/32), Germany (n = 1/32), Mexico (n = 1/32), and South Africa (n = 1/32). More than half of the studies were published in the past 5 years (n = 18/32; 2021 n = 2, 2020 n = 4, 2019 n = 5, 2018 n = 6, 2017n = 1), with most (n = 12/18) using the BCTTv1 taxonomy, which was published in 2013 (Michie et al., 2013). See Supplementary Table 1 for details by individual prospective study.

Across prospective studies a total of 56 of 93 unique BCTs were identified from BCTTv1, plus an additional 5 techniques developed by study authors (Supplementary Table 2). Of the studies that used the BCTTv1 (n = 15), reported number of BCTs ranged from 6 (Burton et al., 2021, Fisher et al., 2019, Marshall et al., 2021, Martin and Murtagh, 2015) to 30 (Fulkerson et al., 2015), with a median number of 14. The most frequently identified BCTs (identified in 9 or more studies) were *4.1 Instruction on how to perform behaviour* (n = 12), *5.1 Information about health consequences* (n = 11), 1.1 *Goal setting (behaviour)* (n = 9), *2.3 Self-monitoring of behaviour* (n = 9), and *6.1 Demonstration of the behaviour* (n = 9) (Michie et al., 2013).

### 3.3 Methods applied in retrospective use of BCT taxonomies

Table 3 presents a summary of retrospective BCT taxonomy applications in childhood obesity prevention. BCTs were most frequently coded from published materials only (n = 12/23), chiefly using the BCTTv1 taxonomy (n = 18/23), by two coders (n = 12/23) or one primary coder and a proportion cross checked (n = 5/23). Coding was predominately independent (n = 16/23), with discrepancies resolved through discussion (n = 14/23) or by consulting a third person (n = 10/23). Approximately half of the studies did not report coder training (n = 12/23). Of those that reported coder training, most reported BCTTv1 online training (n = 10/11). Of the studies that used the BCTTv1 taxonomy, two thirds reported the BCTs with the number and label from the taxonomy (n = 12/18), the remainder reported only BCT labels or different phrasing to describe BCTs (e.g., ‘goal setting’, rather than specifying if it referred to BCT *1.1 Goal setting (behaviour)* or BCT *1.3 Goal setting (outcome)*). Majority of the BCT results were synthesised and reported as narrative summaries (n = 15/23), with few using varied quantitative synthesis approaches. Most retrospective studies were published in the past 5 years (n = 22/23; 2021 n = 3, 2020 n = 7, 2019 n = 5, 2018 n = 3, 2017 n = 4), with most (n = 18/22) using BCTTv1 taxonomy. See Supplementary Table 3 for details by individual retrospective study.

**Table 3:** Summary of BCT taxonomy retrospective applications in childhood obesity prevention (n = 23).

Features of BCT methods^a^		Number of retrospective studies^b^
**Type(s) of intervention materials coded**	Published only Unpublished only Published and unpublished App/website/activity tracker Not reported	12 2 4 2 3
**Taxonomy**	BCTTv1 (incl. adaptions)CALO-RE (incl. adaptions)BCT (Abraham et al 2008) Other/Not reported	18 2 1 2
**Number of coders per study**	1^c^ 2 3 4 Not reported	5 12 1 1 4
**Independence of coders**	Independent Unclear or not reported	16 6
**Process for managing discrepancies in coding**	Discussion between coders Third person/expert consulted^d^	14 10
**BCT training undertaken by coders**	BCTTv1 online training Project specific/other training Not reported	10 4 12
**BCTs synthesis procedure**	Narrative summary Quantitative analysis (e.g., *meta*-regression) Not reported	15 6 2
***Results for studies using BCTTv1***		
**BCTs reported with number and label**^e^	Yes No	12 6
**Average number of BCTs reported per primary study**^f^	Studies reporting BCTTv1:Published materials only (n=8) 5.6 median (range 4.3 to 9) Unpublished materials (only or in addition to published) (n = 4)	
	17.5 median (range 13.9 to 45)	

a Studies could be coded against more than one category for process for managing discrepancies, BCT training undertaken by coders and BCTs synthesis procedure.

b Retrospective studies include systematic reviews, multi-method, secondary analysis, methodology study, exploratory study with content analysis, systematic assessment of applications, intervention development.

c All studies with 1 coder double coded a subset (10 to 22 % double coded or n = 20 double coded) or verified coding by another coder.

d This category captures both the primary approach of consulting a third coder, or only if needed following discussion between coders.

e For studies with results available (n = 18).

f Note this does not differentiate between behaviours targeted, types or intensity of interventions.

Across retrospective studies a total of 80 of 93 unique BCTs were identified from BCTTv1, as well as 4 unofficial BCTs that authors added to the taxonomy for their study (Supplementary Table 4). Of those studies that used the BCTTv1 (n = 15), the median number of BCTs reported per study was 5.6, ranging from 4.3 (Azevedo et al., 2019) to 9 (Johnson et al., 2018) for published reports. The median number of BCTs reported when including unpublished materials was 17.5, ranging from 13.9 (JaKa et al., 2019) to 45 (JaKa, et al., 2021) BCTs. Unpublished materials included items such as manuals for intervention delivery, participant handouts, phone scripts, transcriptions from intervention sessions, and unpublished intervention trial protocols. The number of BCTs identified when coding app/website/activity tracker was a mean of 3.9. Most frequently identified BCTs (used in 10 or more studies) were *3.1 Social support (unspecified)* (n = 15), *1.1 Goal setting (behaviour)* (n = 14), *1.4 Action planning* (n = 14), *1.2 Problem solving* (n = 13), *4.1 Instruction on how to perform a behaviour* (n = 13), *5.1 Information about health consequences* (n = 13), *7.1 Prompts/cues* (n = 13), *2.3 Self-monitoring of behaviour* (n = 13), *6.1 Demonstration of the behaviour* (n = 12), *8.1 Behavioural practice/rehearsal* (n = 12), *12.5 Adding objects to the environment* (n = 12), *3.2 Social support (practical)* (n = 11), *12.1 Restructuring the physical environment* (n = 11), *1.5 Review behaviour goal(s)* (n = 10), and *2.2 Feedback on behaviour* (n = 10) (Michie et al., 2013).

## 4 Discussion

Interventions to change obesity-related behaviours in childhood are complex, and BCT taxonomies provide one approach to unpack this complexity. Prior to this review there was limited literature describing the landscape of research applying BCT taxonomies in childhood obesity prevention. This review has explored how BCT taxonomies have been applied to understand childhood obesity prevention interventions targeting children aged ≤ 12 years and/or their caregivers. We found limited detail in the reporting of BCT taxonomy-related methods, particularly in prospective applications of taxonomies, indicating the need for best practice guidelines for reporting such methods. There was large variation in the number of BCTs reported or identified when using only published materials compared with unpublished materials. Finally, we found five BCTs (*1.1 Goal setting (behaviour), 2.3 Self-monitoring of behaviour, 4.1 Instruction on how to perform behaviour, 5.1 Information about health consequences,* and *6.1 Demonstration of behaviour*) commonly used in childhood obesity prevention interventions, regardless of prospective or retrospective taxonomy application. This review provides a snapshot of the use of BCT taxonomies in childhood obesity prevention and direction to improve applications in this evolving field.

### 4.1 Opportunities to improve reporting of the application of BCT taxonomies

Limited details of methods to identify BCTs were reported in both prospective and retrospective applications of BCT taxonomies. Methods details were particularly scarce in prospective applications. Specific details on BCTs reporting are needed to support evidence syntheses and provide opportunity to understand the impact of specific BCTs on improving obesity-related outcomes. For example, intervention protocols only reporting the names of selected BCTs, with no details of the process undertaken to select the BCTs. Selected BCTs were rarely reported with their corresponding number and label from the taxonomy, and in some instances the BCT taxonomy used was not stated. This is consistent with a previous review of RCTs to improve children’s weight status (2-18y), finding only 6 of 190 studies reported BCTs using standardized definitions (JaKa et al., 2016). Within studies that retrospectively coded BCTs, approximately half did not report coder training and a third did not report BCTs with their corresponding number and label from a taxonomy. Poor methods reporting has implications for our ability to synthesise BCT effectiveness, particularly when there is uncertainty regarding which BCTs were used (i.e., no number and label). It also reduces confidence in the reliability of BCTs coded from existing interventions (e.g., unclear of training, number of coders and independence, types of materials) (Wood et al., 2014).

Poor reporting is not unique to BCT taxonomy applications. For example, a previous review noted limited reporting of certain treatment fidelity components in childhood obesity interventions (JaKa et al., 2016). There have been calls to increase the quality of reporting of behavioural intervention descriptions more broadly with the development of the TIDieR checklist and CONSORT-SPI extension (Grant et al., 2018, Hoffmann et al., 2014). Yet, such guidance does not include reporting of BCT-related processes although an understanding of these components is crucial to unpack the ‘black box’ of intervention core components. We propose similar reporting guidance is needed for retrospective use of BCT taxonomies, potentially as a PRISMA-BCT extension for reporting systematic reviews that code BCTs. In addition, guidance regarding methods for BCT selection and reporting would assist transparency and quality use of taxonomies in prospective applications. Such guidance could supplement coding instructions provided with the BCTTv1 online training for example (BCT Taxonomy v1 Online Training, 2020). Wood and colleagues (2014) have previously provided methods guidance that researchers undertake BCT taxonomy training (BCT Taxonomy v1 Online Training, 2020). A previous review of the different methods to determine BCT effectiveness, could be used to inform recommendations for BCT synthesis (Michie et al., 2018). Finally, methods aspects examined in our review provide direction for expanding guidance to include processes to select BCTs, transparency in coding procedures and clear results presentation, that could align with other intervention reporting guidance (e.g. (JaKa et al., 2016). This will increase confidence in understanding the selection of BCTs in future interventions and reviews. **Box 1** presents preliminary recommendations for reporting BCT taxonomy applications.

Box 1 **: Preliminary recommendations for reporting BCT taxonomy applications in childhood obesity prevention interventions.**

**Table:** 

**Prospective taxonomy applications***(*i.e.*, in the design of interventions)* Name the taxonomy and provide the citation Detail the researchers selecting BCTs, including any BCT training and prior experience Detail the process used to select BCTs in sufficient detail to allow replication, including how each BCT is operationalised in the intervention List of all included BCTs, reported with the corresponding number and label from the taxonomy
**Retrospective taxonomy applications***(*i.e.*, assessing interventions)* Name the taxonomy and provide the citation Detail the researchers coding BCTs, including the number of coders in total, BCT training and prior experience Detail the types of materials used and coding process, including, number of coders per trial, independence of coders, consensus processes List of all identified BCTs, reported with the corresponding number and label from the taxonomy Detail the methods used to synthesis BCT findings

### 4.2 Variation in the number of BCTs identified with types of materials coded

We observed large variation in the number of BCTs identified in retrospective applications of taxonomies when coding only published materials (i.e., publications), compared with unpublished intervention materials (e.g., participant resources) and those reported in prospective studies. The difference in the average number of BCTs identified per study when coding published versus unpublished materials was approximately 12 BCTs. This suggests coding BCTs from publications alone may underestimate the true range of unique BCTs included in an intervention. Publication of intervention protocols and use of Supplementary Files to share all unpublished materials can increase the detail of information available for an intervention, to confidently code BCTs. A broad range of intervention types were eligible for the review so this may reflect differences in BCTs in large, multi-component interventions versus discrete brief interventions, this requires further examination. An alternate explanation may be that when coding unpublished intervention materials that BCTs of lower dose or unintended BCTs are able to be identified. Hence coding of unpublished materials may inflate the number of BCT compared with those planned, or additional BCTs may not add to understanding the behaviour change process.

The median number of BCTs reported by trialists in prospective applications (14 BCTs) was similar to that per individual trial when coding unpublished intervention descriptions, which may support the prior hypothesis that BCTs are under reported in published materials. Developing reporting guidance as proposed above would assist researchers to transparently report the rationale for BCT selection and aid future evidence synthesis by decreasing reliance on coding unpublished materials. However, a larger sample of retrospective studies that have coded unpublished materials is needed to draw firmer conclusions.

### 4.3 Commonly used BCTs in childhood obesity prevention interventions

A range of unique BCTs were selected prospectively (n = 15; 56 of 93) or identified retrospectively (n = 15; 80 of 93) in childhood obesity prevention interventions, when applying the BCTTv1 (Michie et al., 2013). There were several BCTs that were common across study designs, including *1.1 Goal setting (behaviour), 2.3 Self-monitoring of behaviour, 4.1 Instruction on how to perform behaviour, 5.1 Information about health consequences,* and *6.1 Demonstration of behaviour*. Use of such BCTs may suggest common inclusion of intervention components seeking to change individual’s knowledge and motivation. Such BCTs, with the exception of *6.1 Demonstration of behaviour*, have also been commonly reported in systematic reviews of adult obesity prevention or management interventions (Ashton et al., 2020) or interventions targeting dietary intake and/or physical activity in adult populations (Ashton et al., 2019, Howlett et al., 2018, Samdal et al., 2017). In addition, the adult literature has commonly identified *2.2 Feedback on behaviour* and *3.1 Social support (unspecified)* (Ashton et al., 2020, Ashton et al., 2019, Howlett et al., 2018, Samdal et al., 2017). These BCTs were frequently reported in retrospective studies in the current review (BCT 2.2 n = 10; BCT 3.1 n = 13), but less commonly reported in prospective studies (BCT 2.2 n = 3; BCT 3.1 n = 5, respectively). There were only 13 BCTs that were not reported in any study in our review, several of which are unlikely applicable for this research area and target population, such as *8.5 Overcorrection, 10.11 Future punishment, 11.1 Pharmacological support,* and *11.4 Paradoxical instructions*. There were however 20 unique BCTs used in only 1 or 2 prospective studies and 30 unique BCTs reported in only 1 or 2 retrospective studies. The less frequently used BCTs could be incorporated into existing interventions to increase their impact or in new interventions to evaluate their potential to change behaviour in childhood obesity prevention.

### 4.4 Strengths and limitations of the review

Our study is the first to examine the BCT methods used in the prevention of childhood obesity. The review is strengthened by the systematic search and selection process to identify relevant literature. The planned approach was to have two reviewers independently extract data, however, given the number of records identified data extraction was completed by one reviewer with a percentage cross-checked by another reviewer, which is common for scoping reviews and few discrepancies were identified. There were several challenges faced in synthesising the methods used in applying BCT taxonomies in this review, generally because of scarce detail in the methods reported in publications. It is important to consider the findings of this review within the confines of reported intervention detail. For example, in prospective applications training in BCT taxonomies was rarely reported, however researchers may have undertaken such training but not reported this within the intervention publication. There was inherent English language bias despite no search limits being applied for language. We found studies were primarily from the USA, UK and Australia, however recommendations for reported BCT-related methods and results can be applied in other regions. This potential English language or geography bias may be a result of the taxonomies being developed and published in English; future translations of such taxonomies may support uptake of BCT taxonomies in other regions. A final limitation is that data extraction of retrospective studies did not record the frequency of BCT application in the included intervention trials of each study, but rather the total number of unique BCTs and mean number of BCTs identified across trials.

### 4.5 Future directions

The current delve into the application of BCT taxonomies in childhood obesity prevention research has revealed several additional research questions that warrant investigation. Firstly, there is a need to develop best practice guidance for BCT taxonomy application and reporting. Such a process should involve expert consensus involving researchers with expertise in BCT taxonomies and childhood obesity prevention intervention research. Variation in the number of BCTs identified when using different types of intervention descriptions leads to numerous further methods investigations that were out of scope of the current review. It would be helpful to explore the underrepresentation of BCTs in publications or unintended BCTs when examining unpublished materials. If such future research highlights the need to code unpublished materials, guidance will be needed for which types of materials to be prioritised and establish a process for researchers to share open-source materials. An ongoing challenge when using BCT taxonomies relates to the uncertain intensity of each BCT (JaKa et al., 2019), including successful delivery of BCTs by intervention facilitators. Future research is needed to tackle this issue including guidance on how to code and report BCT intensity and dose received by participants. In addition to reporting of BCTs, there is a need for research to extend clarity of reporting of all intervention components that may influence effectiveness (e.g., fidelity (JaKa et al., 2016).

## 5 Conclusions

Our study provides important insight into the methods researchers have used when applying BCT taxonomies in childhood obesity prevention interventions. We found substantial variation in the methods used to apply BCT taxonomies and to report BCT-related methods and results. There was a paucity of detail reported in how BCTs were selected in studies applying BCT taxonomies prospectively. We propose a need for best practice reporting guidance to standardise processes of applying BCT taxonomies and improve the detail reported in publications. We discuss several of the ongoing challenges in how BCT taxonomies are applied and provide direction for future research in this important methods area.

## Funding

This work was supported by the Australian National Health and Medical Research Council (NHMRC) under Ideas Grant TOPCHILD (Transforming Obesity Prevention for CHILDren): Looking into the black box of interventions (GNT1186363). SY is supported by an ARC Discovery Early Career Research Award (DE170100382). RKH is supported by a NHMRC Early Career Fellowship (APP1160419).

## Declaration of Competing Interest

The authors declare that they have no known competing financial interests or personal relationships that could have appeared to influence the work reported in this paper. BJJ, ALS, KEH, AW, SY were authors of one or more of the included studies within this review, however authors were not involved in extracting data for studies of which they were an author.
